# Short-Term Reproduction of Active Movement with Visual Feedback and Passive Movement with a Therapist’s Hands

**DOI:** 10.3390/brainsci14060531

**Published:** 2024-05-23

**Authors:** Hitoshi Oda, Shiho Fukuda, Ryo Tsujinaka, Han Gao, Koichi Hiraoka

**Affiliations:** 1 Graduate School of Rehabilitation Science, Osaka Metropolitan University, 3-7-30 Habikino, Habikino 583-8555, Osaka, Japan; hito_pt23@yahoo.co.jp (H.O.); vqnw01142@ares.eonet.ne.jp (S.F.); gh1823020@outlook.com (H.G.); 2 Graduate School of Comprehensive Rehabilitation, Osaka Prefecture University, 3-7-30 Habikino, Habikino 583-8555, Osaka, Japan; eernvv4487@yahoo.co.jp; 3 School of Medicine, Osaka Metropolitan University, 3-7-30 Habikino, Habikino 583-8555, Osaka, Japan

**Keywords:** pelvic movement, motor learning, rehabilitation, physical therapy, short-term motor memory

## Abstract

Reproducing instructed movements is crucial for practice in motor learning. In this study, we compared the short-term reproduction of active pelvis movements with visual feedback and passive movement with the therapist’s hands in an upright stance. Sixteen healthy males (M age = 34.1; SD = 10.2 years) participated in this study. In one condition, healthy males maintained an upright stance while a physical therapist moved the participant’s pelvis (passive movement instruction), and in a second condition, the participant actively moved their pelvis with visual feedback of the target and the online trajectory of the center of pressure (active movement instruction). Reproduction errors (displacement of the center of pressure in the medial–lateral axis) 10 s after the passive movement instruction were significantly greater than after the active movement instruction (*p* < 0.001), but this difference disappeared 30 s after the instruction (*p* = 0.118). Error of movement reproduction in the anterior–posterior axis after the passive movement instruction was significantly greater than after the active movement instruction, no matter how long the retention interval was between the instruction and reproduction phases (*p* = 0.025). Taken together, active pelvis movements with visual feedback, rather than passive movement with the therapist’s hand, is better to be used for instructing pelvis movements.

## 1. Introduction

Patients with movement disorders practice to-be-learned movements to improve their motor control. Instruction of to-be-learned movements is critically important to how easily or effectively they learn. Possible instructional techniques include the passive movement of the patient’s body part with the therapist’s hands, permitting the patient to perceive and understand the to-be-learned movement via proprioception, and this technique is particularly useful if patients cannot move voluntarily or cannot move correctly.

There are several findings indicating the benefit of passive movement for motor learning. For example, a previous study compared active and passive movement as practices for one to acquire bimanual movement [1]. The frequency of the movement was equally acquired between the movements, although the relative phase of the bimanual movement was better acquired by active movement. Proprioceptive information about the to-be-learned motor task was beneficial for motor learning [2]. The somatosensory system is predominant in the early stages of motor learning [3]. The passive motor experience had an anterograde transfer effect on the subsequent motor execution, whereas no retrograde interference was confirmed [4]. Motor learning is achieved through repetitive holding of the short-term memory of the practiced movement. Accordingly, it is possible to assume that passive movement is beneficial for short-term retention of the memory regarding the instructed movement.

Passive movement of the patient’s body, called “handling,” has been used to improve motor control in patients with central nervous system disorders, and it is exemplified by passive movements of the pelvis. In this example, a therapist rotates a stroke patient’s pelvis with their hands during gait [5]. Pelvis movements are induced or modified by the passive movement with the therapist’s hands during weight shifting or gait [6]. For children with cerebral palsy, the pelvis and legs are supported by the therapist’s legs to maintain stance [7]. According to these examples, in clinical settings, passive movement of the pelvis has been used for guiding the movements who cannot move by themselves or who cannot move correctly. As such, passive movement of the pelvis has been used in clinical settings because it allows patients to understand the to-be-learned pelvis movement even if they cannot move it voluntarily or cannot move it correctly. In spite of these clinical examples, the relative effect of the passive movement of the patient’s pelvis with the therapist’s hands for movement instructions, compared to other techniques, has not been elucidated through empirical research.

Another movement instruction technique is for a therapist to instruct the patient to move actively while providing external feedback. This technique seems to be a more direct way for patients to understand the to-be-learned movement because they actually produce the motor command of the to-be-learned movement. Indeed, previous investigators have shown that active movement instructions were better than passive movement instructions for identifying a target arm position [8], generating less reproduction errors [9], better reproducing a hand position [10], and recalling an object’s location [11]. A possible explanation for these findings is that the proprioceptive system is better tuned to movements created by active muscle contractions rather than passive movement because muscle spindles are subject to central fusimotor control [10].

Despite those previous findings, comparing active movement instructions with visual feedback to passive movement instructions with the therapist’s hands is still crucial because there are differences between these conditions other than just the difference between the presence or absence of active muscle contractions. First, for participants to understand the to-be-learned movement via active movements, the instructor must provide the patients with target information and external feedback of the movement. In contrast, in passive movement with someone else’s hands, participants understand the movement via proprioceptive sensations rather than external feedback. Second, when the movement is instructed via the passive movement of the participant’s body with someone’s hands, there is a tactile sensation caused by the holding of the body part with the therapist’s hands. The findings in the past studies investigating the different effects of the active and passive movement instructions did not take into account the influence of visual feedback and tactile sensation. To address this possibility, in the present study, we compared these two movement instruction techniques (active and passive pelvis movements) with respect to the reproduction of the instructed pelvis movement to determine which instructional method may be preferred for introducing movements in physical therapy. The somatosensory system is predominant during the early stages of motor learning [3]. Pelvis movement to a target appearing at the different locus in each trial mimics the situation in the early stage of motor learning. Thus, we hypothesize that short-term reproduction of the passive movement of the pelvis with the therapist’s hand is better than that of the active movement with visual feedback.

## 2. Materials and Methods

### 2.1. Participants

The present study was an intervention study. Our participants were sixteen healthy males (M age = 34.1, SD = 10.2 years). We chose only male participants because the therapist in this study was male, and we sought to minimize arousing sexual reactions to pelvic touch in cross-sex patient–therapist interactions. Our sample size was based on sample sizes in previous studies that found a significant effect of vision or tactile sensation on body sway during a quiet stance [12] (*n* = 16) and found a significant difference in the reproduction of active and passive movements [8] (*n* = 16). According to the number of the participants in those previous studies, we considered that 16 participants were sufficient to detect the significant difference in the reproduction error of the active and passive pelvis movement instructions in stance. The participants had no history of neurological or orthopedic diseases. All participants gave written informed consent for study participation before engaging in this experiment. The experimental protocol was approved in advance by the ethics committee of Osaka Metropolitan University.

### 2.2. Apparatus

**Figure 1:**
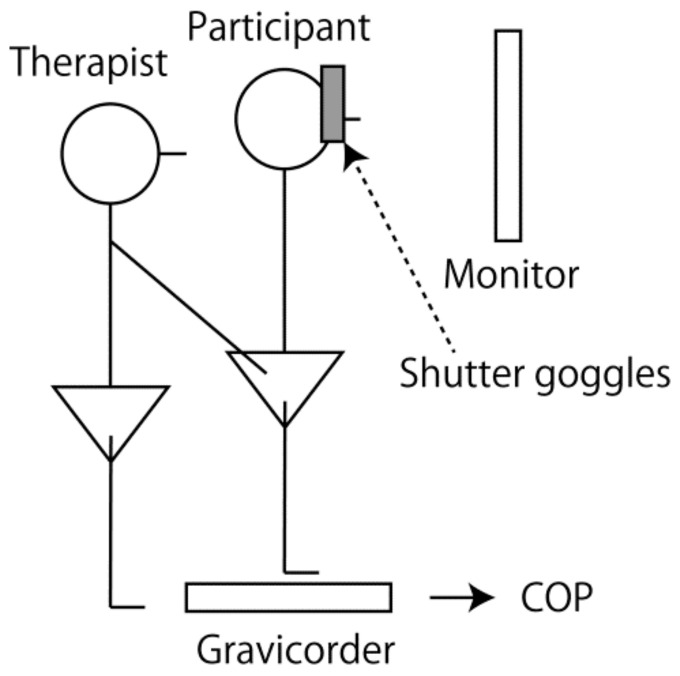
Experimental setup. A participant maintains standing over the support surface of the gravicorder, and a therapist stands behind him. A monitor is placed in front of the participant and therapist. The participant wears shutter goggles that occlude their vision temporarily. COP—center of pressure.

The experimental setup is shown in Figure 1. A gravicorder, measuring the participants’ center of pressure (COP), was placed on the ground (1G06/I-B, Nihon Denshi Sanei, Tokyo, Japan). Strain gauges measuring the COP were placed at each corner of the support surface of the gravicorder. From the vertical force measured in those strain gauges, we calculated the COP. The change in the output level with 0.1 V indicates a 1 cm deviation of the COP in this gravicorder. The amplified analog signals from the gravicorder were digitized by an A/D converter (PowerLab/8sp; ADInstruments, Colorado Springs, CO, USA) at a 1 kHz sampling rate and stored in a PC. The participants wore liquid shutter goggles that occluded their vision (T.K.K.2275; Takei Kiki, Tokyo, Japan).

### 2.3. Target and COP Cursor

**Figure 2:**
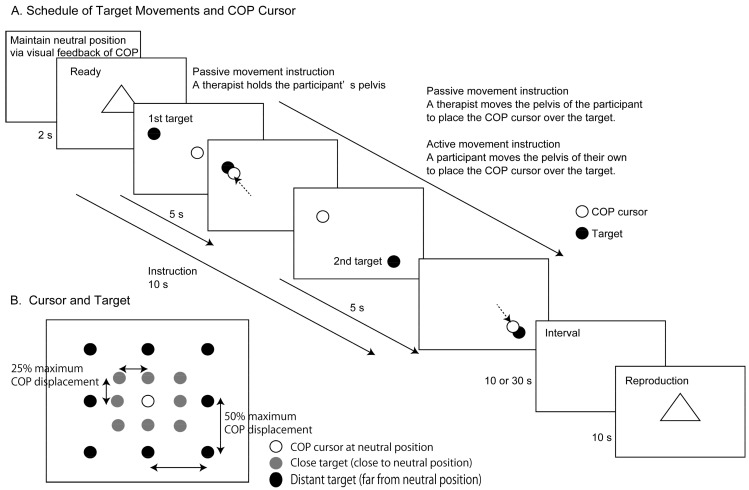
The schedule of the target movements and COP cursor on the monitor. Display (**A**) and cursor indicating target COP and cursor indicating online COP position (**B**). A participant moves his own pelvis (active movement instruction) or a therapist moves the participant’s pelvis (passive movement instruction) so that the COP cursor is at the target in the instruction phase, and the participant reproduces the instructed pelvis movement in the reproduction phase (**A**). A target appears at 1 of 16 sites (1st target) and then appears at another site (2nd target) in the instruction phase (**B**). Arrows indicate movement of a COP cursor.

A target and COP cursor are shown in Figure 2B. A monitor showing the target and COP cursor was placed 50 cm in front of the participants. The target, a red circle outlined in black, indicated the endpoint of the COP of the instructed pelvis movement. The COP cursor, a white circle outlined in black, indicated the online COP. The participants first stood on the support surface of the gravicorder and maintained a quiet stance, with their feet placed in the position where the COP cursor was at the center of the monitor (neutral position). The upward movement of the cursor on the monitor indicated a forward displacement of the COP, and the rightward movement of the cursor indicated a rightward displacement of the COP.

### 2.4. Sensitivity of COP Cursor

The COP displacement during the inclination of the body at maximal effort in stance is individually different due to the participants’ inter-individual differences in body size and body inclination ability. Thus, we adjusted the sensitivity of the cursors for each participant so that the distance of the target from the neutral position relative to the distance from the target during the maximal inclination of the body would be equal for all participants.

Before conducting the experiment, we had the participants engage in a preliminary trial to determine the sensitivity of the COP cursor in the monitor. The participants maintained a stance on the gravicorder with their legs closed and arms next to the trunk. We opened the shutter goggles to permit vision. A triangle appeared on the monitor for 5 s. While the triangle was presented, the participants inclined their bodies forwards and backwards once, and then inclined their bodies rightwards and leftwards once at maximum effort without leaving the feet from the support surface of the gravicorder. The sensitivity of the COP was set to reflect the degree to which the COP deviated from the neutral position, with 100 pixels on the display corresponding to 25% of the maximal COP deviation of this trial.

### 2.5. Procedure

Preliminarily, an experimenter confirmed that the cues, targets, and cursor on the monitor were visible for the participants and therapist. Four phases were sequentially proceeded in order of (a) the ready phase, (b) the instruction phase, (c) the retention interval phase, and (d) the reproduction phase in each trial (Figure 2A). In the ready phase, participants first maintained a quiet stance in the neutral position. In the instruction phase, they received a pelvis movement instruction via active or passive movement. In the retention interval phase, they maintained a memory of the instructed movement. In the reproduction phase, they reproduced the instructed pelvis movement. Two target distances (close and distant targets) were applied to each of the two retention intervals (10 and 30 s). There were eight trials in each instruction condition. Thus, four conditions (close target with a 10 s interval, close target with a 30 s interval, distant target with a 10 s interval, and distant target with a 30 s interval) were applied to each instruction condition over a total of 64 trials. The Instruction Conditions, Intervals, Target Distances, and Target Directions were randomly assigned in each trial.

The time course of the visual cues on the monitor is shown in Figure 2A. Participants were informed about the instruction condition before each trial. Before the experiment, an experimenter asked the participants to memorize the instructed pelvis movement for reproducing the instructed movement. Participants maintained a quiet stance on the gravicorder and gazed at the monitor. A male physical therapist, who knew the experimental design and had 18 years of experience in geriatric physical therapy, stood behind the participant. Before the ready phase, the participants received online visual feedback of the COP and made an effort to locate the COP at the neutral position.

A triangle, indicating the beginning of the trial, appeared for 2 s when the COP was stable at the neutral position (ready phase). In this phase, the participants maintained a quiet stance and directed their eyes to the monitor. In the passive movement instruction condition, the physical therapist held the lateral aspect of the participant’s pelvis with his hands. In the active movement instruction condition, the physical therapist maintained a stance behind the participant without touching the participant.

In the instruction phase next to the ready phase, a target and COP cursor appeared on the monitor for 10 s. In the first half of this phase (first 5 s), the target was presented at a first target position (first target). In the second half of this phase (last 5 s), the target appeared at a second target position (second target) in one out of eight directions relative to the neutral position (i.e., forward, backward, leftward, rightward, leftward and forward, leftward and backward, rightward and forward, and rightward and backward directions; Figure 2B). The target on the monitor was at a locus 100 or 200 pixels away from the neutral position in both the medial–lateral and anterior–posterior axes. The target at the locus 100 pixels away from the neutral position corresponded to the displacement of the COP from the neutral position with 25% of the maximum COP deviation (close target). The target at the locus 200 pixels away from the neutral position corresponded to the displacement of the COP from the neutral position with 50% of the maximum COP deviation (distant target).

In the instruction phase of the passive movement instruction condition, the shutter goggles were closed so that the target and COP cursor were invisible to the participants but visible to the therapist. The therapist moved the participant’s pelvis with his hands to place the COP cursor at the target.

In the instruction phase of the active movement instruction condition, the shutter goggles were opened. The participants voluntarily moved their pelvis so that the COP cursor was at the target. In the active movement instruction condition, an experimenter asked the participants to “move the pelvis so that the open circle is positioned at the red circle” before each trial.

After this phase, the target and COP cursor disappeared for 10 or 30 s, and the participants maintained their stance at the neutral position (retention interval phase). The participants were not told in advance what the length of the retention interval phase would be before each trial. This interval was used to investigate the participant’s short-term motor memory retention in accordance with the protocol in previous studies [13,14,15]. The therapist maintained a stance behind the participant without touching the participant’s pelvis.

At the end of the retention interval phase, a triangle appeared for 10 s (reproduction phase). The participants attempted to reproduce the pelvis movement experienced in the instruction phase. An experimenter asked the participants to “move the pelvis as perceived in the previous pelvis movement when a triangle appears” before beginning the experiment. In this phase, the shutter goggles were opened so that the participants could view the triangle. The therapist maintained a stance behind the participant without touching the participant’s pelvis.

### 2.6. Data Analysis

We analyzed the participants’ COP in the instruction and reproduction phases. First, we examined whether the difference in the COP between the two instruction conditions (active and passive movement instructions) in the instruction phase was significant. This analysis was conducted to confirm that the COP in the instruction phase was not significantly different between the two instructions to rule out the possibility that the error or bias in the reproduction of the movement was due to the difference in the COP between the instruction conditions in the instruction phase. Second, we calculated the average difference in COP between the instruction and reproduction phases (constant error; CE). The CE represented the across-trial average bias towards a particular direction [16]. Accordingly, the CE represented the reproduction bias of the instructed pelvis movement. Finally, the absolute value of the CE (absolute error—AE) represented the across-trial average error, irrespective of the error’s direction [17,18]. Therefore, the AE represented the reproduction error of the instructed pelvis movement. The COP measured in the present study represented the displacement of not only of the pelvis but also the other body parts. Accordingly, the reproduction error of the instructed pelvis movement in the present study was regarding the error in the deviation of the body parts linked to the pelvis movement while the therapist or participant intended to move the pelvis.

The average difference in the COP between the two instruction conditions in the instruction phase was statistically compared with zero using a one-sample *t*-test with a Bonferroni adjustment of alpha. This test was performed to examine whether the COP in the instruction phase was significantly different between the instruction conditions. We used a repeated measures four-way analysis of variance (ANOVA) to examine the effect of the Instruction Condition (two levels), Target Distance (two levels), Retention Interval (two levels), and Target Periods (two levels). This four-way repeated-measures ANOVA was conducted to examine the main effects and examine the interaction between the main effects. We used partial eta squared to reflect effect sizes. The result of Greenhouse–Geisser’s correction was reported whenever Mauchly’s test of sphericity was significant. If there was a significant interaction between the main effects, tests of the simple main effects were conducted. If there was a significant main effect of the inter-trial retention interval, multiple post-hoc comparison tests (Bonferroni’s test) were conducted. We set the alpha level to 0.05. We used Excel-Toukei 2010 ver. 1.13 (Social Survey Research Information, Tokyo, Japan) for our statistical analysis, and we expressed descriptive data in terms of means and the standard error of the mean.

## 3. Results

### 3.1. COP Differences in the Instruction Phase

**Figure 3:**
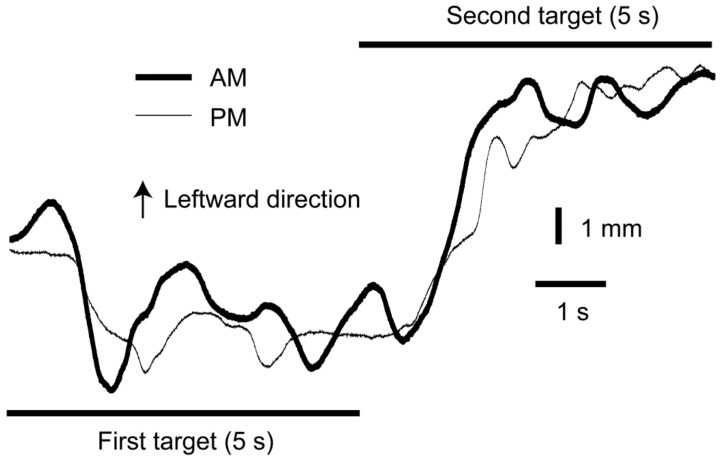
Example of COPx traces in the instruction phase. Two traces are similar in the instruction phase. PM—passive movement instructional method; AM—active movement instructional method.

An example of the COPx traces in the instruction phase is shown in Figure 3, and the average difference in the COP in the instruction phase between the two instruction conditions (active and passive pelvic movement) is shown in Table 1. The difference in the average COPx in the instruction phase between the two instruction conditions was not significantly different from zero in any of the Target Phases, Target Directions, or Target Distances (one-sample *t*-test with a Bonferroni adjustment of alpha). Similarly, the differences in the average COPy in the instruction phase between the instruction conditions was not significantly different from zero in any of the Target Phases, Target Directions, or Target Distances (using a one-sample *t*-test with a Bonferroni alpha adjustment), except for the leftward and forward, leftward and backward, and rightward and backward directions in the close first target. That is, in most of the conditions, the COP in the instruction phase was not significantly different between the instruction conditions.

### 3.2. Reproduction Error in the Medial–Lateral Movement Axis

**Figure 4:**
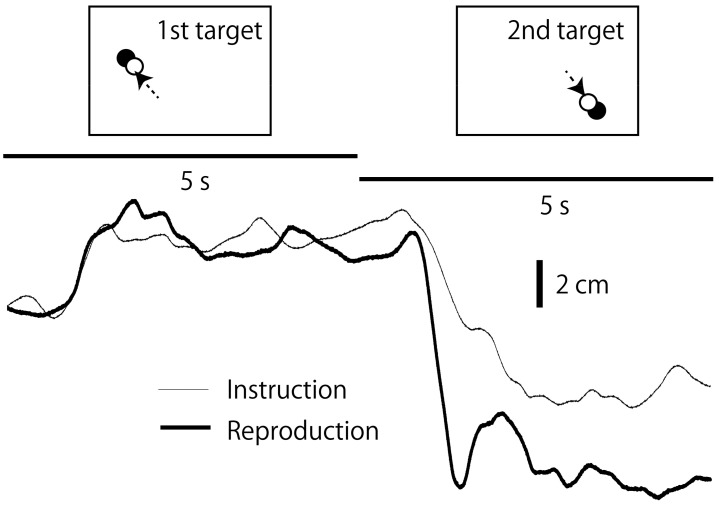
Example of COPx traces in the instruction and reproduction phases of the passive movement instruction condition. For the 2nd target, a large difference in the traces between the instruction and reproduction phases is present, whereas it is not for the 1st target. Arrows indicate movement of a COP cursor.

**Figure 5:**
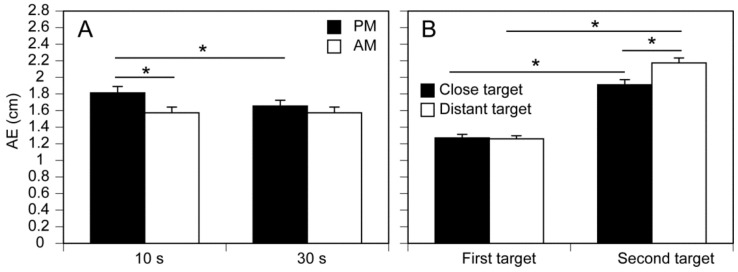
Simple main effect on the AE in the COPx. The simple main effects of the instruction condition and retention interval (**A**) and that of the target distance and retention interval (**B**) are shown. Bars indicate the mean, and error bars indicate the standard error of the mean. Asterisks indicate a significant difference (*p* < 0.05). AM: active movement, PM: passive movement, and AE: absolute error.

An example of the COPx traces is shown in Figure 4, and the mean AE values are shown in Figure 5. There was a significant main effect of the Instruction Condition (F(1, 15) = 12.246, *p* = 0.003, η^2^p = 0.449), Target Distance (F(1, 15) = 5.556, *p* = 0.032, η^2^p = 0.270), Retention Interval (F(1, 15) = 17.804, *p* < 0.001, η^2^p = 0.543), and Target Period (F(1, 15) = 153.494, *p* < 0.001, η^2^p = 0.911) on the AE (reproduction error). There was a significant interaction between the Instruction Condition and the Retention Interval (F(1, 15) = 17.517, *p* < 0.001, η^2^p = 0.539). The test of simple main effects revealed that the AE in the passive movement instruction condition was significantly greater than that in the active movement instruction condition at the 10 s Retention Interval (F(1, 20) = 23.352, *p* < 0.001), but not at the 30 s Retention Interval (F(1, 20) = 2.677, *p* = 0.118). The test of simple main effect revealed that the AE in the 10 s Retention Interval was significantly greater than that in the 30 s Retention Interval for the passive movement instruction condition (F(1, 30) = 35.310, *p* < 0.001) but not for the active movement instruction condition (F(1, 30) = 0.003, *p* = 0.958). The simple main effects of the Instruction Condition and Retention Interval are shown in Figure 5A.

There was a significant interaction between the Target Distance and Target Period (F(1, 15) = 15.204, *p* < 0.001, η^2^p = 0.503), but there was no significant simple main effect of the Target Distance on the first target (F(1, 26) = 0.030, *p* = 0.863). The test of the simple main effect revealed that the AE for the distant target was significantly greater than for the close target in the second target (F(1, 26) = 16.912, *p* < 0.001). The test of the simple main effect revealed that the AE for the second target was significantly greater than for the first target both for the close (F(1, 24) = 78.936, *p* < 0.001) and distant targets (F(1, 24) = 161.525, *p* < 0.001). The simple main effects of Target Distance and Retention Interval are shown in Figure 5B.

### 3.3. Reproduction Error in the Anterior–Posterior Movement Axis

**Figure 6:**
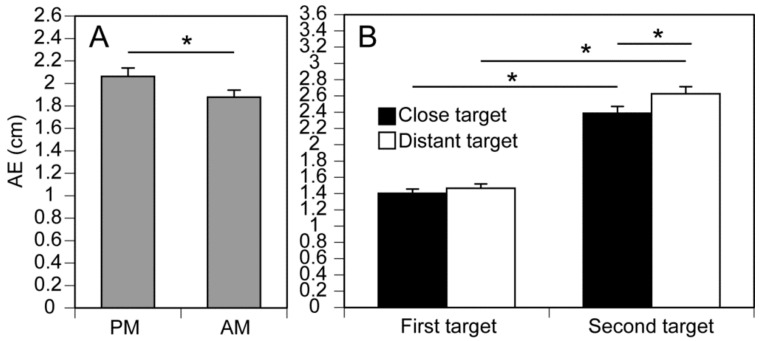
Main effect of the instructional method (**A**) and simple main effects of the Target Period and Target Distance (**B**) on the AE of COPy. Bars indicate the mean, and error bars indicate the standard error of the mean. Asterisks indicate a significant main effect (**A**) or simple main effects (**B**) (*p* < 0.05). AM: active movement, PM: passive movement, and AE: absolute error.

There was a significant main effect of the Instruction Condition (F(1, 15) = 6.205, *p* = 0.025, η^2^p = 0.293), Target Distance (F(1, 15) = 4.909, *p* = 0.043, η^2^p = 0.247), and Target Period (F(1, 15) = 233.573, *p* < 0.001, η^2^p = 0.940) for AEs (reproduction errors) in the anterior–posterior movement axis. AEs with the passive movement instruction condition were significantly greater than with the active movement instruction condition. The main effect of the Instruction Condition is shown in Figure 6A. There was no significant main effect of the Retention Interval (F(1, 15) = 0.128, *p* = 0.726, η^2^p = 0.008).

There was a significant interaction between the Target Distance and Target Period (F(1, 15) = 8.566, *p* = 0.010, η^2^p = 0.363). The test of the simple main effect revealed that the AE for the distant target was significantly greater than for the close target for the second target (F(1, 21) = 10.282, *p* = 0.004). The test of the simple main effect revealed that the AE for the second target was significantly greater than for the first target for both the close (F(1, 20) = 166.029, *p* < 0.001) and distant targets (F(1, 20) = 230.942, *p* < 0.001). The simple main effects of the Target Distance and Target Period are shown in Figure 6B.

### 3.4. Reproduction Bias

**Figure 7:**
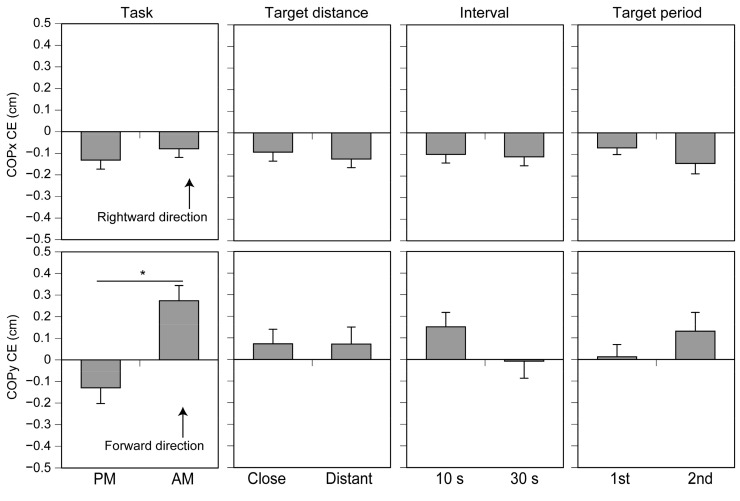
Main effects on the CE. Bars indicate the mean, and error bars indicate the standard error of the mean. Asterisks indicate a significant main effect (*p* < 0.05). AM: active movement, PM: passive movement, and CE: constant error.

There was no significant main effect of the Instruction Condition (F(1, 15) = 0.588, *p* = 0.455, η^2^p = 0.038), Target Distance (F(1, 15) = 0.588, *p* = 0.455, η^2^p = 0.038), Retention Interval (F(1, 15) = 0.030, *p* = 0.864, η^2^p = 0.002), or Target Period (F(1, 15) = 1.631, *p* = 0.221, η^2^p = 0.098) on the CE (reproduction bias) in the COPx. There was a significant interaction between the main effects of the Instruction Condition, Target Distance, and Target Period (F(1, 15) = 6.939, *p* = 0.019, η^2^p = 0.316), but pairwise comparisons did not reveal a significant difference between any pairs of means. There was a significant main effect of the Instruction Condition (F(1, 15) = 13.463, *p* = 0.002, η^2^p = 0.473) on the CE in the COPy, with the CE in the active movement instruction condition being significantly greater than in the passive movement instruction condition (Figure 7). This means that the forward bias of the movement reproduction in the active movement instruction was greater than in the passive movement instruction. There were no significant main effects of the Target Distance (F(1, 15) = 0.000, *p* = 0.984, η^2^p = 0.000), Retention Interval (F(1, 15) = 3.118, *p* = 0.098, η^2^p = 0.172), and Target Period (F(1, 15) = 0.901, *p* = 0.358, η^2^p = 0.057) on the CE in the COPy, and there were no significant interaction between the main effects. The main effects on the CE are shown in Figure 7.

## 4. Discussion

In this study, we compared the reproduction errors in participants’ pelvis movements between two movement instructions: active movement with visual feedback of the body sway and passive movement with the hands of a therapist. The reproduction error was smaller when movements were instructed via the active movement with visual feedback, but the time course of this difference was dependent on the body sway direction.

### 4.1. Reproduction Error

On the one hand, the AE of the COPx displacement in the passive movement instruction condition was greater than in the active movement instruction condition when the retention interval between the instruction and reproduction phases was 10 s. On the other hand, this significant difference disappeared when the retention interval was 30 s. Accordingly, the reproduction error of body sway in the medial–lateral axis was smaller within 10 s after the end of the instruction for the active movement instruction with visual feedback. This finding is in line with previous findings that reproduction of the active movement was better than that of the passive movement [8,9,10,11]. It has been shown that the somatosensory system predominantly acts in the early stage of motor learning [3], but the present finding suggests that the short-term reproduction of the instructed pelvis movement does not process as during the early phase of motor learning.

Reproduction of the instructed movement is achieved through holding and recalling the memory of the instructed movement. Accordingly, our finding means that the active movement instruction with visual feedback of the cursor and target on the monitor better helped participants memorize the to-be-learned movement when reproduction was nearly immediate (within 10 s after the instruction). One possible view for the better reproduction in the active movement instruction is that participants better recalled the instructed movement when the content of the memory involved the internal motor command of the pelvis movement, while when instructed via passive movement by the therapist’s hands, participants did not recall the motor command because the active movement was absent in the instruction phase.

There was no significant difference in the reproduction error between the instruction conditions when reproduction was made 30 s after the instruction. On the one hand, the reproduction error was not significantly different between the intervals in the active movement instruction condition. On the other hand, the error in the 10 s interval was worse than that in the 30 s interval in the passive movement instruction condition. Based on these findings, the better movement reproduction performance after the active than passive movement instruction at the 10 s interval may be better explained by poor movement reproduction after the passive movement instruction at this interval.

It has been well established that short-term motor memory is decayed over time [19,20,21]. More importantly, memory decay occurred 10 s after the end of the target force’s production [14]. Accordingly, one may speculate that the change in reproduction over time is due to the decay of memory. However, the present finding did not support this view. Reproduction was better as the retention interval was increased from 10 to 30 s in the passive movement instruction condition (i.e., memory became better over time) in the present study. Thus, the decrease in the reproduction error from a 10 to 30 s interval in the passive movement instruction condition is not explained by the time decay of the short-term motor memory.

One possible explanation for the decrease in the reproduction error from a 10 to 30 s interval in the passive movement instruction condition is that the passive movement instruction distracted the participant’s effort to recall the instructed movement when asked to reproduce the movement within 10 s after the instruction. In the passive movement condition, a therapist moved the pelvis of the participant with their hands, and thus, the tactile sensation of holding the pelvis with the therapist’s hands must have been generated [22]. Accordingly, a tactile sensation induced by the holding of the participant’s pelvis with the therapist’s hands may have distracted the memory process of the passive movement within 10 s after the instruction.

In contrast to the finding in the body sway in the medial–lateral axis, the reproduction error in the passive movement instruction condition was greater than in the active movement instruction condition for the anterior–posterior movement axis, even at 30 s after the end of the instruction phase. This means that the memory process of the pelvis movement was worse in the passive instruction condition for this axis, regardless of the retention interval. The control of the body position in the medial–lateral axis is different from that in the anterior–posterior axis in an upright stance; the control of the anterior–posterior body sway is mediated by the ankle, but in the medial–lateral axis, it is mediated by the hip [23]. Thus, different reproduction errors between these body sway axes in the present study may be due to these different body part strategies. In the passive movement condition, a therapist held the participant’s body part around the hip (i.e., the lateral aspect of the hip) with their hands, and this may have had a greater distraction effect on the hip strategy in the medial–lateral axis.

### 4.2. Target Periods

The reproduction error of the second target was greater than that of the first target. One possible view is that this finding is related to a capacity limitation of working memory [24,25,26,27]. Recall of short-term motor memory is worsened as the required memory for holding information increases. Thus, the greater reproduction error of the second target may be due to the overtaxed memory of the instructed movement to the second target. However, our view is unlikely to be true. Participants had to recall the instructed movement to both the first and second targets before reproducing movement to the first target, but they only needed to recall instructed movement to the second target before reproducing movement to the second target. That is, their memory size must have been smaller before recalling the movement to the second target. This would seem to conflict with the memory capacity limitation explanation for a worse reproduction of movement to the second target.

It has been well established that inserting irrelevant movements between the practice and reproduction of the task distracts the recall process for the reproduction. For example, the reproduction error after a 12 s retention interval was greater as the number of interpolated irrelevant movements during the retention interval increased [28]. On the one hand, in the present study, the memorized movement to the second target in the instruction phase was inserted between the memorizing and recalling processes of the movement to the first target. On the other hand, the recalling process of the instructed movement to the first target in the reproduction phase was inserted between the memorizing and recalling processes of the movement to the second target. Based on this, the present finding is explained by the view that the recall process of one movement, rather than the memorizing process of that, greatly distracts the recall process of the subsequent movement. Another possible explanation is the difference in the start position of each target phase. The participants began at the neutral position when reproducing movements to the first target, but they began at the endpoint of the first target’s reproduction for the movement to the second target. Thus, memorizing, holding, or recalling the pelvis movement may have been more difficult when the movement starts from the end of the preceding movement.

### 4.3. Reproduction Bias

The CE in the active movement instruction condition was significantly greater than that in the passive movement instruction condition. This means that the memory of the body position was biased forward when reproducing the instructed pelvis movement after the active movement instruction condition, compared with the passive movement instruction condition. Two explanations are possible for this finding. One is that the memory of the motor command caused a forward bias of the memory of the active movement instruction. Another is that the tactile sensation of the pelvis caused a backward bias of the memory of the passive movement instruction. Further studies are needed to elucidate which view is correct.

### 4.4. Clinical Implications

A precise understanding of the optimal methods for movement instructions is crucial for practicing the to-be-learned movement. Long-term learning of the practiced movement is achieved through repetition of its correct reproduction. We found that active movements with visual feedback were generally better than the passive movement instruction. Yet, therapists must instruct movements without active movement for patients who cannot move independently. In addition, to use active movements for instructions, there must be instrumentation for providing target feedback that can be difficult to obtain or implement in clinical settings. More importantly, patients with motor disorders have difficulty producing movements, causing difficulty in the use of the active movement for instructing the to-be-learned movement. Accordingly, passive movement with the therapist’s hands remains a necessary practical choice for instructing movements.

Our finding that the reproduction of the first target was better than that of the second target suggests a distraction of the recall process of the second instructed movement induced by the preceding recall process of the first movement. Thus, in clinical settings, especially for patients with difficulty memorizing instructed movements, therapists should use discrete movements, delivered singly and separately rather than sequential movements when instructing to-be-learned movements.

### 4.5. Limitations and Directions for Further Research

The purpose of the present study was to compare the effects between the active and passive movements of the pelvis for instructing the movement. However, motor control or memory process may be somewhat different between healthy humans and patients with central nervous system disorders. Thus, it is not certain that the present finding on healthy humans is applicable for patients with motor disorders who have difficulty in motor control. Further studies on patients with motor disorders are needed to elucidate whether the present findings are applicable for clinical settings. There was a significant interaction between the Target Distance and Target Period on the reproduction error in both COPx and COPy. This was an extraordinary finding, and we have no idea how to explain this finding. Further studies are needed on this finding.

## 5. Conclusions

The reproduction error 10 s after the passive instruction was significantly greater than after the active movement with visual feedback about body sway along the medial–lateral axis, but this difference disappeared 30 s after the instruction. The reproduction error of the body sway along the anterior–posterior axis for the passive movement instruction condition was significantly greater than that for the active movement instruction, no matter the retention interval between the instruction and reproduction phases. We concluded that the tactile sensation in the passive movement distracted reproduced movement along the medial–lateral axis immediately after the instruction but not after a 30 s delay, and that the better reproduction after the active movement instruction along the anterior–posterior axis demonstrated a clear advantage to active movement with the visual feedback instruction over the passive movement instruction for pelvic movements.

## Figures and Tables

**Table 1:** Differences in COP between passive and active movement in the instruction phase.

			L (cm)	R (cm)	F (cm)	B (cm)	L + F (cm)	R + F (cm)	L + B (cm)	R + B (cm)
COPx										
	1st	Close	0.0 ± 0.1	0.0 ± 0.1	0.1 ± 0.1	0.0 ± 0.1	0.0 ± 0.1	0.0 ± 0.1	−0.1 ± 0.1	0.0 ± 0.1
		Distant	−0.1 ± 0.1	0.0 ± 0.1	0.1 ± 0.1	0.0 ± 0.1	−0.1 ± 0.1	−0.1 ± 0.1	0.0 ± 0.1	0.0 ± 0.1
	2nd	Close	−0.1 ± 0.1	0.2 ± 0.1	0.0 ± 0.1	0.0 ± 0.1	0.0 ± 0.1	0.2 ± 0.1	−0.1 ± 0.2	0.0 ± 0.1
		Distant	−0.3 ± 0.1	−0.1 ± 0.1	−0.1 ± 0.1	−0.1 ± 0.0	0.0 ± 0.1	0.0 ± 0.1	−0.3 ± 0.1	0.2 ± 0.1
COPy										
	1st	Close	0.3 ± 0.1	0.2 ± 0.1	0.4 ± 0.1	−0.1 ± 0.1	0.3 ± 0.1 *	0.4 ± 0.1	−1.1 ± 0.2 *	0.8 ± 0.1 *
		Distant	0.0 ± 0.1	0.0 ± 0.1	0.1 ± 0.1	−0.1 ± 0.0	0.0 ± 0.1	0.2 ± 0.1	−0.1 ± 0.1	−0.1 ± 0.1
	2nd	Close	0.2 ± 0.1	0.2 ± 0.1	0.3 ± 0.1	0.2 ± 0.1	0.3 ± 0.1	0.4 ± 0.1	−0.1 ± 0.1	0.1 ± 0.1
		Distant	−0.3 ± 0.1	0.0 ± 0.1	−0.2 ± 0.1	0.0 ± 0.1	0.0 ± 0.1	0.2 ± 0.1	−0.4 ± 0.1	−0.1 ± 0.1

L; leftward movement, R; rightward movement, F; forward movement, B; backward movement; * *p* < 0.05 (significantly different from zero; one-sample *t*-test with Bonferroni adjustment of alpha).

## Data Availability

The datasets used or analyzed in this study can be obtained from the corresponding author upon reasonable request. The data are not publicly available due to privacy issues.
